# Psychosocial characteristics of victims of special fraud among Japanese older adults: A cross-sectional study using scam vulnerability scale

**DOI:** 10.3389/fpsyg.2022.960442

**Published:** 2022-07-25

**Authors:** Daisuke Ueno, Masashi Arakawa, Yasunori Fujii, Shoka Amano, Yuka Kato, Teruyuki Matsuoka, Jin Narumoto

**Affiliations:** ^1^Department of Psychiatry, Graduate School of Medical School, Kyoto Prefectural University of Medicine, Kyoto, Japan; ^2^Second Organized Crime Division, Kyoto Prefectural Police Headquarters, Kyoto, Japan

**Keywords:** special fraud, fraud victimisation, fraud vulnerability, scam vulnerability, solitude, frequency of outing, older adults, scam vulnerability scale

## Abstract

Despite the police preventing special fraud victimisation of older adults, both the number of cases and the amount of damage have remained high in Japan. ‘Special fraud’, in Japan, is a crime in which victims are tricked by fraudsters who through phone or postcards impersonate the victims’ relatives, employees and other associates, to dupe the victims of their cash or other valuables. The number of recognised cases of special fraud has been turned to increase in 2021. Although police or consumer affairs administrations have been conducting all-encompassing enlightenment or public education for prevention, it is also necessary to reach out to those who are vulnerable to fraud. In this study, we determine the psychosocial characteristics of victims of special fraud in Japanese older adults. We analysed the age, gender, education, residential status, household satisfaction, risk perception and scam vulnerability scale of 56 older adults aged 60 years or older (mean age: 79.34 ± 7.51 years, 49 women) who had been victims of special fraud and 99 older adults aged 60 years or older (mean age: 77.73 ± 5.69 years, 61 women) who had never been victims of special fraud. The study found that the victimised older adults were more likely to be females who live alone and go out less frequently than the non-victimised older adults. The total scores of the scam vulnerability scale were higher among the elderly victims of special fraud compared to those who had never been scammed, suggesting that the psychosocial characteristics of victims of special fraud among older adults are being female, living alone, going out infrequently, having high confidence against fraud victimisation and responding quickly to phone calls and unknown visitors. Therefore, government agencies or family members should take care of older women who meet these characteristics to reduce their contact with fraudsters.

## Introduction

In recent years, numerous incidents of fraud with its associated economic loss have been reported in Japan, with cases of special fraud being frequently reported. The term ‘special fraud’ in Japan refers to crimes committed under the disguise of being relatives or employees of public institutions by telephone or postcard (sealed envelope). Criminals trick victims into believing that they can receive cash, cash cards, or that they can receive refunds for medical expenses, and then encourage them to transfer money to the criminals’ accounts through ATMs. This includes extortion and stealing cash cards by swapping them when an opportunity arises (National Police Agency, 2022a). To diversify the methods of special fraud and to strengthen countermeasures against damage, the NPA has eliminated the conventional distinction between ‘remittance fraud’ and ‘no-remittance special frauds’ and has introduced 10 types of fraud (Table 1): ‘It’s Me’ fraud, bank account fraud, billing fraud, refund fraud, advance-fee loan fraud, financial investment fraud, lottery fraud, romance fraud, other special frauds, and bank card fraud and theft (Metropolitan Police Department, 2021).

**Table 1:** Categories of Japanese fraud (special fraud) tactics.

Categories	Description
‘It’s Me’ fraud	The person claiming to be a relative or other family member says, ‘I left my bag behind, I found a cheque in it, or I need some money’, and then he or she asks you to transfer to the designated account.
Bank account fraud	The person claiming to be a police officer, bank official, and so on, says ‘Your account has been used for criminal purposes and we need to replace your cash card’. Alternatively, the person says ‘You overpaid for medical expenses. We will take care of it here, and I will go and get your cash card’. Then, he or she will ask you for your PIN number and attempt to defraud you and using your cash card.
Billing fraud	The person sends an e-mail or postcard (sealed envelope) to the customer informing him or her that ‘there are unpaid fees for pay sites or consumption charges, and if you do not pay them by the end of the day, we will take you to court’, and then he or she asks you to transfer to the designated account or buys a prepaid card and ask you for your PIN number.
Refund fraud	The person claiming to be a government official says ‘You overpaid for medical, tax, or insurance expenses’, and then he or she makes you believe that he or she is processing your refund at the ATM, whereas your money is transferred to a designated account. Although this tactics is similar to the Bank account fraud, it differs because the fraudster does not meet the victim but gives instructions over the phone.
Advance-fee loan fraud	In fact the person does not loan, but fraudster makes you believe that you can obtain a loan easily from him or her, and then he or she defrauds the loan applicants by telling the applicant that a deposit is required.
Financial investment fraud,	The person gives you false information about unlisted stocks or expensive goods that have no value at all, and makes you believe that you can gain the profits if you purchase them. Then he or she defrauds you of your cash once you pay for the purchase.
Lottery fraud	The person who posts a magazine to you or sends you an e-mail making you believe that you can win money or dividends in exchange for teaching gambling winning methods. Then he or she defrauds you by making you pay a registration or information fee.
Romance fraud	The person who posts a magazine to you or sends you an e-mail of someone who wishes to introduce you to men or women, and then defrauds you by making you pay a membership registration fee or a deposit.
Other special frauds	The special fraud that does not fall into any of the listed categories.
Cash card fraud and theft	The person claiming to be a police officer, bank official, or government official says ‘Your cash card is being used illegally so I will make it unusable’, and substitutes and steals your cash card. Although the tactic is similar to the Bank account fraud and Refund fraud, it differs from them because the intention of the fraudster is to steal your cash cards.

The number of recognised cases of special fraud in Japan has been decreasing continuously since 2018 but rapidly increased in 2021 to 14,498 (+948 cases or +7.0% compared to the previous year). Additionally, the total amount of damage caused by special fraud was 28.20 billion yen (−0.32 billion yen, −1.1% compared to the previous year) It has been decreased for 7 years since 2014 (National Police Agency, 2022a). However, both the number of recognised cases and the total amount of damage have remained high. The number of recognised cases of special fraud among the aged 65 and over was 12,724 (−1,137 cases or + 9.8% compared to the previous year), and the ratio of victims among older adults to the total victims excluding corporate victims was 88.2% (+2.4 points compared to the previous year). Thus, the number of elderly victims of special fraud is increasing (National Police Agency, 2022a). There is a more prevalent rate of consumer fraud among older adults in Japan compared with the United States (Ross et al., 2014). The ratio of female victims among older adults is approximately 68.7% (+2.6 points from the previous year), and it is also characteristic that many older women are victims of special fraud (National Police Agency, 2022a). As a result, the number and amount of fraud victims in Japan have remained high, with the majority of victims being older adults. In addition, the amount of damage per case of special fraud in 2021 is 2,020,000 yen (−182,000 yen, −8.2% compared to the previous year), which is much higher than the amount of damage per case of theft (909,000 yen as of 2019). Therefore, fraud prevention measures focusing on older adults are very important to prevent economic loss.

Lichtenberg et al. (2013) surveyed 31,000 participants (mean age of 65.76 ± 8.54 years, 61.9% female, 4.5% victims of fraud) in the Health and Retirement Study (HRS). They investigated the incidence of fraud and psychological factors among these participants. The results showed that victims of fraud tended to have fewer years of education, higher levels of depression, and lower levels of household satisfaction. Moreover, Lichtenberg et al. (2016) studied 4,661 participants who are over 50 years old (mean age of 67.73 ± 9.28 years as of 2008, 61.2% female, 4.3% fraud victims) and discovered that younger age, more years of education, higher levels of depression were associated with more fraud. Lichtenberg et al. (2016) explained that younger age predicted fraud victimisation because people are more likely to engage in high-risk financial transactions when they are worried about their retirement. The results also revealed that there may be indirect factors such as the inability to manage household finances on one’s own, going out less frequently, and having fewer opportunities to contact fraud perpetrators in older adults. Concerning years of education and fraud victimisation, it is also possible that the education system relates to the direct proportion of the number of years of education and age.

Van Wyk and Mason (2001) conducted a telephone survey on the relationship between opportunities for social participation and fraud victimisation (including consumer problems) among 400 people aged 18 years or older. They found that if the younger participants had more social connections, then they had a higher frequency of fraud victimisation. Additionally, younger participants are more likely to take risks with their investments, and risk preferences are more frequent in fraud victimisation. Moreover, Alves and Wilson (2008) reported that older adults who had experienced telemarketing fraud tended to be divorced/separated, while fraud victims tended to live alone, suggesting that residential status may be related to fraud victimisation.

In this study, we examined the relevance of fraud vulnerability as a psychosocial characteristic associated with fraud victimisation. Scales measuring fraud vulnerability include a scale on telemarketing fraud (James et al., 2014), a scale using scenarios of fraudulent situations (Zhang et al., 2017), and a scale on behavioural, cognitive, and emotional characteristics found in fraud victims (Ueno et al., 2021). James et al. (2014) developed a five-item, seven-point telemarketing fraud vulnerability scale (e.g. confidence that one will not be scammed) based on the findings of the American Association of Retired Persons (American Association of Retired Person, 1999) and the U.S. Financial Industry Regulatory Authority (FINRA) developed the Risk Meter based on the FINRA Investor Education Foundation (2007). Gamble et al. (2014) assessed fraud vulnerability based on whether they were registered with the National Do Not Call Registry operated by the US Federal Trade Commission (2019). Zhang et al. (2017) assessed an 18-item, four-point elderly fraud risk scale (e.g. would you like to participate in free health counselling services offered in the city?) using scenarios related to common economic fraud tactics such as healthcare, online banking, investment, financial management, and emergency assistance requests. Zhang et al. (2017) listed the psychological characteristics related to fraud vulnerability among older adults, such as easier following blindly, a stronger desire for profit, easier belief in authority, easier belief in superstition, lack of information channels, and not wanting to cause trouble for others. Akiyama (2013) cited the following as the tactics of perpetrators: emotional arousal (inciting anxiety and fear, making people feel happy, etc.); time urgency (pressuring people to respond immediately, such as right now, today, etc.); confirmation bias (using assumptions and preconceptions); and authority (pretending to be a policeman or a lawyer to prevent people from pointing out things). Button et al. (2014) reported fraudster techniques committed against online fraud victims, including grooming, authority, legitimacy, visceral appeals, embarrassing the victim, or pressure and coercion.

Although the characteristics of fraud vulnerability have been identified, only a few quantitative studies have demonstrated them. One of the reasons for this is the problem with the measurement scale. The fraud vulnerability scale (James et al., 2014) has limited fraudulent tactics, such as telemarketing fraud, and it is difficult for older adults with cognitive decline to answer scenarios of fraudulent situations (Zhang et al., 2017). Although James et al. (2014) and Zhang et al. (2017) reported that fraud victims were more vulnerable to fraud using the total score of the scale as the dependent variable, the authors did not conduct analyses on individual items.

The psychosocial characteristics of elderly victims of fraud in the U.S. are younger age, more years of education, lower life satisfaction, exposure to more opportunities for social participation, higher risk preferences, living alone, and higher vulnerability to fraud. In this study, we examined and clarified the psychosocial characteristics of victims of special scams in Japanese older adults. In Japan, although there is a survey on the awareness of victims and attempted victims of ‘It’s Me’ fraud, such as their knowledge of fraud tactics and confidence in not being scammed (National Police Agency, 2018), to the best of our knowledge, there is no scientific study on the psychosocial characteristics of elderly victims of special fraud in Japan.

To prevent fraud victimisation, police or consumer affairs administrations have been conducting enlightenment or public education for fraud prevention and distributing telephone recording devices in Japan. Although enlightenment or public education for prevention by the local administrations may highly contribute to fraud prevention, the effectiveness of these efforts is unclear (Mears et al., 2016). Moreover, Mears et al. (2016) reported that although fraud prevention education efforts would likely help educated older adults in managing their finances more effectively and in using the internet and the internet shopping prudently, fraud vulnerability, such as fraud targeting and victimisation does not drive older adults to seek out information or assistance with fraud prevention. There seems to be a limit to countermeasures that can be taken by individuals through conventional all-encompassing educational activities, and it is necessary to act against those who are vulnerable to fraud in the community. In Japan, the Consumer Safety Act was amended in 2015, which requires the establishment of regional councils for ensuring consumer safety (Consumer Affair Agency, 2015). The regional councils for ensuring consumer safety are established in 391 local governments. Such regional councils could share information on those who are vulnerable or fraud victims, and it is possible to take countermeasures by focusing on those who are more vulnerable to fraud. Consequently, it is important to identify the psychosocial characteristics of those who are vulnerable to fraud.

The purpose of this study is to clarify the psychosocial characteristics, including fraud vulnerability, of elderly victims of special fraud and older adults who have never experienced fraud, to promote the prevention of such fraud. To clarify fraud vulnerability with fraud victimisation, we used the Scam Vulnerability Scale (Ueno et al., 2021) to compare the scores of each item between the elderly victims of fraud and older adults who have never experienced fraud.

## Materials and methods

### Participants

Fifty-six older adults aged 60 years or older who had been victims of fraud (victims group; mean age 79.34 years, SD = 7.51, 49 females) and 99 older adults aged 60 years or older who had never been victims (non-victims group; mean age 77.73 years, SD = 5.69, 61 females) were included in the study. Participants in the non-victims group were included in the previous report (Ueno et al., 2021). Victims submitted a report to a police station in Kyoto Prefecture from September 1, 2018, to August 31, 2019. They had also submitted written consent to the ‘Survey for Creating a Vulnerability Test to Prevent Fraud Victimisation among Older Adults’ conducted by the Special Fraud Prevention Office of the Kyoto Prefectural Police Headquarters from September 1, 2019, to November 30, 2019. The selection criteria in the victims’ group were those who were 60 years old or older and could give written informed consent. Two participants who had been victims of fraud or consumer problems were excluded from the non-victims group. The selection criteria in the non-victims group were 60 years old or older and scored a Clinical Dementia Rating (CDR; Hughes et al., 1982) of 0.5 or less or a Mini-Mental State Examination (MMSE; Folstein et al., 1975) of 26 or less, but participants who were able to give written informed consent or a substitute, were independent in daily living, and had an MMSE score of 27 or higher were able to give written informed consent (Ueno et al., 2021). Exclusion criteria were a history of mental illness, head injury, drug or alcohol abuse, intellectual disability, and significant visual or hearing impairment. A power analysis assumed an uncorrelated t-test with the dependent variable being the Scam Vulnerability Scale score and the independent variable being the presence or absence of victimisation, with an effect size of 0.50, an alpha error of 0.05, power (1−β) of 0.8, and an n-ratio of the two groups of 2. The required sample size was 48 victims and 96 non-victims. This study was approved by the Medical Ethics Review Committee of Kyoto Prefectural University of Medicine (ERB-C-1845).

### Questionnaire survey items

#### Years of education

To measure the number of years of education, we asked the participants to fill in the number of years of education, including primary education.

#### Type of residence

A 5-point nominal scale (1–5) was used to measure the type of residence. The options were: (1) living alone (no other living relatives), (2) living alone (other living relatives), (3) a couple only, (4) living with a child and (5) other. We distinguished those living alone whether they had other living relatives or not. This is because older adults who lived alone and had no other relatives, were less likely to be exposed to identity theft scams, and their vulnerability to scams could be affected by not consulting their families.

#### Household satisfaction

A five-point ordinal scale (1–5) was used to measure household satisfaction as the level of life satisfaction. The options were: (1) not satisfied at all, (2) not satisfied, (3) somewhat satisfied, (4) very satisfied, and (5) completely satisfied.

#### Frequency of going out

To measure the frequency of going out as an opportunity for social participation, we used a four-point ordinal scale (1–4). The options were: (1) once or more every day, (2) once every two or 3 days, (3) once a week and (4) rarely. The range of outings was defined as outside the premises of the home or apartment, not including short-distance and short-time outings such as throwing away trash in the neighbourhood.

#### Decision-making

Three items related to decision-making were established to measure risk preference. The first was the emotional framing of medicine choices. Respondents were asked to choose one of four options (medicine A = 1; medicine B = 2, both to the same extent = 3, do not know = 4) in response to the following question. The second was risk framing in gaining situations. For the following questions, participants were asked to choose one of two options (lottery for A = 1, lottery for B = 2). The third was risk framing in losing situations. They were asked to choose between two options (lottery C = 1, lottery D = 2) for the following questions.

- Emotional framing of medicine choices: ‘Which medicine is more dangerous?’

Medicine A: Out of 100 people, 5 people’s diseases got worse, but the rest got better. (Negative framing).

Medicine B: Out of 100 people, 95 people’s diseases got better, but the rest got worse. (Positive framing).

- Risk framing in gaining situations: ‘Which lottery do you choose?’

Lottery A: There is an 80% chance that you will get 4,000 yen, but a 20% chance that you will get nothing.

Lottery B: You have a 100% chance of getting 3,000 yen.

- Risk framing in losing situations: ‘Which lottery do you choose?’

Lottery C: There is an 80% chance of losing 4,000 yen, but a 20% chance of losing nothing.

Lottery D: There is a 100% chance of losing 3,000 yen.

#### Scam vulnerability scale

In this study, we used the Ueno et al. (2021) Fraud Vulnerability Scale, a scale of cognitive, behavioural, and emotional characteristics related to fraud vulnerability. The items of the Scam Vulnerability Scale (Ueno et al., 2021) used in this study are shown below. The Fraud Vulnerability Scale comprises nine items, and a Likert scale comprises four points (3 = *Applicable* to 0 = *Not applicable*). They were used for the following statements designed to measure the susceptibility to fraud, which can be answered by older adults with cognitive decline. Three points per item indicate a higher vulnerability to fraud. It was developed based on interview data with four victims of fraud, the telemarketing fraud vulnerability scale (James et al., 2014), and awareness surveys on fraud vulnerability conducted by the police. Ueno et al. (2021) compared the total score of the Scam Vulnerability Scale between cognitively declining and cognitively preserved older adults and found no statistically significant difference between the two groups. In addition, Ueno et al. (2021) calculated the Cronbach’s alpha coefficient, which was the highest for the six items except for items 1, 2, and 4. However, because the number of items on this scale is small and the Cronbach’s alpha coefficient was low, to clarify the characteristics of those who experienced frauds related to items 1, 2, and 4, nine items were used in this study.

- I am confident that I will not be scammed.
- If someone I do not know visits, I do not listen to them. (reverse item).
- Even if I am dissatisfied with my situation, I am overpowered by my opponent.
- I pick up the phone as soon as I get a call.
- I am interested in tempting offers.
- Even if I think the other person’s story is suspicious, I think in a good direction.
- If someone I do not know talks to me in a strong tone, I will be frightened.
- If someone praises or gives special treatment to me, I will be happy.
- I feel anxious about talking to my family and friends about money because it is likely to lead to me losing their trust.

### Procedure

After informed consent was obtained, participants completed a questionnaire. The participants in victims’ group were asked to fill in the Scam Vulnerability Scale after and before victimisation in the same survey.

### Statistical analysis

To clarify the sociodemographic and fraud vulnerability characteristics of those who had experienced fraud, we clarified the differences between those who had experienced fraud (victims group) and those who had not (non-victims group). Specifically, age, years of education, household satisfaction, and Scam Vulnerability Scale scores were tested with an uncorrelated t-test; household satisfaction and frequency of going out were tested with a Mann–Whitney U-test. A χ^2^ test was conducted for the three items of gender, type of residence, and decision-making. Logistic regression analysis with increasing likelihood ratio variables was conducted to clarify the characteristics of sociodemographic and fraud vulnerability related to fraud victimisation. The objective variable was fraud victimisation and the explanatory variables were age, gender, years of education, residential status, household satisfaction, frequency of going out, nine items and total score (for victims’ groups using before victimisation) of the Scam Vulnerability Scale, and three items of risk preference. Statistical analysis was performed using IBM SPSS Statistics 25, with a significance level of *p* < 0.05.

## Results

### Characteristics of the damage situation

In 2019, the number of victims of special fraud in Kyoto Prefecture was 206, 166, aged 65 or older. In this study, we cooperated with 56 elderly victims of special fraud (about 33.7%). Table 2 shows the characteristics of the victim group regarding the special fraud tactics, the method of initial contact by the fraudster, and the identity given by the fraudster. Regarding the special fraud tactics, bank account fraud was the most common (58.9%), followed by billing fraud (23.2%), cash card fraud and theft (8.9%), ‘It’s Me’ fraud (3.6%), advance-fee loan fraud (1.8%), lottery fraud (1.8%), and unknown (1.8%). As for the method of initial contact with the scammers, the telephone was the most common method (about 75%), followed by short message service (about 8.9%), e-mail (about 7.1%), mail (about 3.6%), a website (about 3.6%), and fax (about 1.8%). As for the identity of the fraudsters, government officers accounted for 35.7% of the total, followed by bank officers at 14.3%, police officers at 8.9%, service clerks at 7.1%, and relatives at 1.8%. Officers of public institutions such as government offices, police, and ministries accounted for 46.4% of the total. The number of unknown cases is high because the fraudsters did not identify themselves or did not remember their identities.

**Table 2:** Frequency of the special fraud tactics; the method of first contact with the fraudster; the identity given by the fraudster to victims.

Frequency of characteristics in victims	Frequency	%
Special fraud tactics		
Bank account fraud	33	58.9
Billing fraud	13	23.2
Cash card fraud and theft	5	8.9
‘It’s Me’ fraud	2	3.6
Advance-fee loan fraud	1	1.8
Lottery fraud	1	1.8
No damage amount	1	1.8
Method of first contact with the fraudster		
Telephone	42	75.0
Short message service	5	8.9
Electrical mail	4	7.1
Postal mail	2	3.6
Website	1	1.8
Facsimile	1	1.8
Unknown	1	1.8
Identity given by the fraudster		
Public sector staff	20	35.7
Banking staff	8	14.3
Police officers	5	8.9
Retail clerks	4	7.1
Ministry officials	1	1.8

*The total does not add up to 100% because the second decimal place is rounded up*.

### Characteristics of sociodemographic

The sociodemographic and decision-making characteristics of the victims and non-victims’ groups are shown in Table 3. There were more females, fewer years of education, more solitary, and went out less frequently in the victims group compared with the non-victims group. There were no statistically significant differences between the two groups on age, household satisfaction, and the three items related to decision-making. Statistical details are shown in Table 3.

**Table 3:** Characteristics of psychosocial and decision-making in victims and non-victims of special fraud among older adults.

	Victims	Non-victims	Test statistic and value of *p*	Effect size
Age^1^	77.73 (5.69)	79.34 (7.51)	*t* (91) = 1.40, 0.166	*r* = 0.15
Sex^2^	Female (87.5%)	Female (61.6%)	*χ**^2^* (1) = 11.63, 0.001^**^	*φ* = 0.27
Year of education^1^	11.95 (2.85)	13.12 (2.51)	*t* (153) = −2.65, 0.009^**^	*r* = 0.21
Type of residence^2^	Alone^3^ (47%)	Couple (24%)	*χ**^2^* (5) = 17.79, 0.003^**^	*φ* = 0.34
Household satisfaction^1^	3.33 (0.92)	3.45 (0.83)	*t* (153) = 0.78, 0.435	*r* = 0.06
Frequency of going out^2^	Every day (46%)	Every day (74%)	*U* = 1904, 0.001^***^	*r* = −0.31
Decision-making				
Affective frame^2^	Medicine A (29%)	Medicine B (30%)	*χ**^2^* (3) = 1.85, 0.604	*φ* = 0.11
Gaining frame^2^	Lottery B (80%)	Lottery B (88%)	*χ**^2^* (1) = 1.60, 0.215	*φ* = 0.09
Losing frame^2^	Lottery C (57%)	Lottery C (70%)	*χ**^2^* (1) = 2.48, 0.115	*φ* = 0.13

*Standard deviation or percentage is listed in parentheses*.

1 *Average is listed,*

2 *Mode is listed,*

3 *Other living relatives,*

** *p* < 0.01;

*** *p* < 0.001.

### Characteristics of fraud vulnerability

The means and standard deviations on the Scam Vulnerability Scale for the victims and non-victims’ groups are shown in Table 4. The victims group before victimisation showed that the total score, item 1, item 2, and item 4 were significantly higher than the non-victims group. The victims group before victimisation showed that item 1, item 2, item 3, item 4, item 5, item 6, and item 7 were higher than the victims group after victimisation. The non-victims group showed that total scores were higher tendency, items 3, 5, and 6, were significantly higher than the victims group after victimisation, respectively. In addition, the victims group after victimisation showed that item 1 was significantly higher than the non-victims group. Statistical details are shown in Table 4.

**Table 4:** Characteristics of the fraud vulnerability scale in victims and non-victims of special fraud among older adults.

	Victims		Non-victims	Test statistic and value of *p*			Effect size		
	Before	After		Before × After	Before × Non-victims	After × Non-victims	Before × After	Before × Non-victims	After × Non-victims
Item 1	2.20 (1.07)	1.82 (1.28)	1.37 (0.85)	*t* (55) = 2.13, 0.037^*^	*t* (95) = 4.94, 0.0001^***^	*t* (83) = 2.34, 0.022^*^	*r* = 0.28	*r* = 0.45	*r* = 0.25
Item 2	1.43 (1.26)	0.89 (1.23)	0.89 (1.01)	*t* (55) = 3.14, 0.003^*^	*t* (95) = 2.74, 0.004^**^	*t* (97) = 0.02, 0.983	*r* = 0.39	*r* = 0.27	*r* = 0.01
Item 3	0.86 (0.10)	0.46 (0.87)	0.88 (0.58)	*t* (55) = 3.04, 0.004^*^	*t* (76) = −0.15, 0.864	*t* (83) = −3.18, 0.001^***^	*r* = 0.38	*r* = 0.02	*r* = 0.33
Item 4	2.16 (1.19)	1.52 (1.36)	1.35 (0.93)	*t* (55) = 3.97, 0.0001^***^	*t* (93) = 4.38, 0.0001^***^	*t* (85) = 0.80, 0.375	*r* = 0.47	*r* = 0.41	*r* = 0.09
Item 5	0.50 (0.93)	0.20 (0.55)	0.61 (0.49)	*t* (55) = 2.61, 0.012^*^	*t* (73) = −0.79, 0.355	*t* (103) = −4.62, 0.001^***^	*r* = 0.33	*r* = 0.09	*r* = 0.41
Item 6	0.75 (0.98)	0.34 (0.82)	0.65 (0.52)	*t* (74) = 4.34, 0.001^**^	*t* (73) = 0.74, 0.391	*t* (153) = −2.86, 0.005^**^	*r* = 0.05	*r* = 0.09	*r* = 0.23
Item 7	0.91 (1.10)	0.77 (1.10)	0.84 (0.60)	*t* (55) = 1.24, 0.220	*t* (74) = 0.46, 0.605	*t* (74) = 0.45, 0.657	*r* = 0.16	*r* = 0.05	*r* = 0.05
Item 8	1.00 (1.04)	0.80 (1.03)	0.93 (0.56)	*t* (55) = 1.85, 0.070	*t* (73) = 0.47, 0.583	*t* (73) = −0.84, 0.402	*r* = 0.24	*r* = 0.05	*r* = 0.10
Item 9	0.64 (1.03)	0.77 (1.11)	0.87 (0.65)	*t* (55) = −0.93, 0.358	*t* (80) = −1.48, 0.143	*t* (77) = −0.62, 0.536	*r* = 0.12	*r* = 0.16	*r* = 0.07
Total	10.45 (4.53)	7.57 (3.30)	8.71 (3.59)	t (55) = 5.18 0.001^**^	*t* (94) = 2.63, *p* = 0.009^**^	t (153) = −1.83 0.070	r = 0.57	r =. 26	r = 0.15

*Standard deviation or percentage is listed in parentheses*.

* *p* < 0.05;

** *p* < 0.01;

*** *p* < 0.001.

### Factors associated with fraud victimisation

Based on the odds ratios, the first factor influencing fraud victimisation was being female (OR = 3.52, 95% = 1.15, 10.84), followed by higher scores on item 1 of the Scam Vulnerability Scale (OR = 3.24, 95% = 1.98, 5.29), followed by less frequent outings (OR = 2.78, 95% = 1.51, 5.11), followed by higher scores on item 2 of the Scam Vulnerability Scale (OR = 2.58, 95% = 1.64, 4.07), followed by fewer years of education (OR = 0.82, 95% = 0.69, 0.98).

## Discussion

### Status of fraud damage

In this study, of the special fraud tactics, bank account fraud was the most common at 58.9% of the total, and there is a lot of fraud victimisation aimed at ripping off bank books, bank cards, and credit card. Moreover, in 2021, the coronavirus disease pandemic led to an increase in the number of special fraud that do not require contact with the fraudsters. These fraud tactics include billing fraud and refund fraud, in which a person buys an electronic money card, gives the card number to the fraudsters, or is tricked into believing that money will be returned by operating an ATM. Because there is a trend in the increase and decrease of fraud tactics, it is important to develop countermeasures and create awareness according to appropriate trends for each type of special fraud. As for the method of first contact with a fraudster, the telephone is the most common method, indicating that everyone has a chance of being contacted by a fraudster. In the first call, the fraudsters continue to talk to prevent the target person from consulting someone from hanging up the phone or calling in a specific area so that their fellow fraudsters can go to the target person’s home immediately. Therefore, older adults should reduce contact with fraudsters through phone calls. Moreover, the most common identity of the fraudsters is officers of public institutions such as government offices, police departments, and ministries (46.4% of the total). This tendency suggests that it is easy to be victimised by using the credibility and authority of public institutions.

### Fraud victimisation and sociodemographic characteristics

In this study, we clarified the psychosocial characteristics of elderly victims compared to non-victims of special fraud. Regarding social characteristics, this study showed that the elderly victims of the special fraud were more likely to be female, to live alone and to go out less frequently compared to the non-victims. National Police Agency (2022a) reported that many victims of special frauds were women. Moreover, our finding is consistent with Alves and Wilson’s (2008) report that many victims are older adults who live alone. However, our findings are not consistent with Alves and Wilson’s (2008) report that demonstrated that fraud victims tend to have more opportunities to participate in social activities. In Van Wyk and Mason’s (2001) study, young people were also included in the participants, therefore, this may not necessarily be a characteristic of older adults. In the present study, the highest percentage of fraud victims (75%) were first contacted by telephone (including those visited by a person pretending to be a government or bank officer after confirming their presence by telephone). This reflects the tendency of fraud victims to go out less frequently and stay at home more often. In the future, it is necessary to develop an environment or framework where older adults living alone and staying at home for a long time can easily consult with family members and government agencies.

### Fraud victimisation and fraud vulnerability characteristics

The victims group before victimisation showed that the total score of the scale, items 1, 2, and 4, were higher than the non-victims group. Item 1 was ‘I am confident that I will not be scammed’. Item 2 was ‘When strangers visit me, I try not to listen to them (reversal item)’, and item 4 was ‘When the phone rings, I pick up the receiver immediately’. It is reasonable that item 1 is higher in the victims group before victimisation than in the non-victims group because 95.2% of the victims of ‘It’s Me’ fraud answered that they would not be scammed (National Police Agency, 2018). Confidence in not being scammed is also included in James et al.’s (2014) telemarketing scam vulnerability scale and it is a well-known risk for scam victimisation and scam vulnerability. Items 2 and 4 include the behavioural trait of responding to phone calls or visits from a scam perpetrator, suggesting that victims may easily contact the scam perpetrator. Therefore, to avoid being victimised, it is important not to accept visits or phone calls from strangers, even from people claiming to be from public institutions. Instead, older adults should say no and consult with their families or public institutions. Fraud perpetrators will try to prevent you from talking to others, so it is crucial that you do not accept visits or phone calls from strangers with whom you do not usually have contact, especially if you are confident that you will not be scammed.

The victims group before victimisation showed that the total scores and each item except for items 7, 8, and 9 were higher than victims group after victimisation. Item 7 was ‘I get frightened when a stranger speaks to me in a strong tone’, item 8 was ‘I get happy when I am praised or receive special treatment’, and item 9 was ‘I am worried about losing the trust of my family and friends when I ask for advice about money. These items included emotional characteristics such as fear, happiness, and anxiety, and it became clear that the vulnerability to fraud characteristics related to emotions did not change before and after the victimisation. Kircanski et al. (2018) reported that both older and younger people in the high positive and negative affect arousal groups had higher levels of fraud vulnerability, such as purchase intention and credibility, to fraudulent virtual advertisements than those in the low arousal group. In other words, the reason why fraud vulnerability characteristics related to emotions were not related to fraud victimisation in this study suggests there is a possibility that not the kind of emotion (i.e. valence level), but the intensity of emotion (i.e. arousal level) is related to fraud victimisation.

In addition, the victims group after victimisation showed that there was no statistically significant difference in the total scores of the scales, but higher in item 1, and lower in items 3, 5, and 6 compared to the non-victims’ group. Item 3 was ‘Even if I am dissatisfied, the other person pushes me’, item 5 was ‘I am interested in a good story’, and item 6 was ‘Even if I think the other person’s story is suspicious, I think in a good direction’. These results suggest that after being victimised by a fraud, older adults are more cautious than the non-victimised group to avoid being taken in by what others are saying. Since these characteristics were obtained after the actual victimisation, it is unclear to what extent they can be changed through crime prevention education, but it may be important to include the prevention education of special frauds.

The score for item 1 was the highest in the victim group before victimisation among groups. According to the National Police Agency (2018), 95.2% of the victims and 85.1% of attempted victims of ‘It’s Me’ fraud thought that they would not be victimised. The National Police Agency (2018) does not show statistically significant differences in confidence in not being victimised by scams among victims, attempted victims, and non-victims groups of ‘It’s Me’ fraud, however, our study suggests that confidence in not being victimised by fraud is an outstanding characteristic of fraud victims. The psychology behind this confidence in not being victimised may be due to cognitive biases such as normality bias and optimism bias. The normality bias is a type of cognitive bias in which we believe that emergencies are unlikely to happen to us to maintain our normality because they are threatening to us. Optimism bias is a cognitive bias that makes us overconfident in our abilities. These cognitive biases do not affect fraud victims in older adults individually, but optimism bias is interrelated with normality bias, and they may evaluate themselves as being fine (optimism bias) because they are normal (normality bias). Older adults have been reported to have an optimism bias compared to younger adults, and if they underestimate the incident rate of a negative event, it shows optimism bias, they are less likely to revise their estimation rate after being presented with the actual incidence rate (Chowdhury et al., 2014). Therefore, optimism bias may be related to the confidence of not being a victim of fraud, and future studies need to clarify the correlation between optimism bias and fraud victimisation and vulnerability.

Moreover, the score of item 1 tends to be high even after the victimisation, and it may be difficult to change the confidence in not being a victim of fraud. However, National Police Agency (2018) showed a group of self-spotted victims answered less that ‘I think that fraud was none of their business’, but much more that ‘I know the fraud tactics well’, ‘I always consult someone’, and ‘I have already taken countermeasures’. McKenna et al. (2020) proposed that it is important to provide information and increase awareness about the victimisation and countermeasures on primary prevention and coping with victimisation when it does occur. From the viewpoint of fraud prevention, instead of transforming their confidence in not being a victim of fraud, they need to be aware of the crisis that fraud can happen to everyone and promote knowledge of fraud tactics, avoid making decisions on their own, and take immediate countermeasures to build a basis of confidence in not being a victim of fraud.

### Factors related to fraud victimisation

Among the factors related to fraud victimisation examined in this study, the order of influence was being female, having a higher score on item 1 on the Scam Vulnerability Scale (I am confident that I will not be victimised by fraud), less frequent outings, having a higher score on item 2 on the Scam Vulnerability Scale, and years of education. Therefore, priority needs to be given to older adults who possess psychosocial characteristics and take countermeasures to prevent becoming a victim of special fraud.

### Limitations

We need to be careful in the definition of fraud in this study because this study focuses on ‘special fraud’ in Japan. Because, in many countries, as in Japan, the criminal acts of fraud are often covered by a complex combination of legislation, the definitions and typologies of fraud and statistical methods may be different. There are a few definitions and typologies which are cyber-dependent crimes and cyber-enabled crimes or comprehensive taxonomy (Button and Cross, 2017). In fact, cyber-dependent fraud or card not present fraud, phishing scams and fraudulent sales in cyber-enabled crimes are not but mass marketing frauds and romance frauds included in special fraud in Japan. English and Wales Crime Survey had reported fraud victims including cyber-crimes since 2016, these statistics supported that fraud crime is not necessarily falling, but changing its form (Button and Cross, 2017). In Japan, fraud in cyber-crimes has increased since 2019 (National Police Agency, 2022b), and not to become too preoccupied with the legal definitions, a comprehensive definition and typology that also considers the situation of victimisation and victim’s characteristics.

We need to be careful in interpreting the results of the before victimisation because the fraud victims were asked to remember their answers before victimisation when they were victimised. If a prospective cohort study design provides questions about fraud victimisation, as in Lichtenberg et al. (2013, 2016), it is possible to analyse factors associated with fraud victimisation retrospectively after the victimisation has occurred. However, in Japan, the incidence of elderly victims of special fraud was 0.032% in 2020 (11,556 victims per 36.17 million population aged 65 or older), and self-reported victimisation may be even lower than the number of recognised cases. Scheibe et al. (2014) conducted a study on participants who had been victims of actual fraud to examine whether prior warning can prevent victims of telephone fraud. In this study, the dependent variable was whether a laboratory assistant with telemarketing experience made a fake fundraising solicitation phone call to the participants and subsequently agreed to receive a parcel with an invoice and details. Thus, from an ethical point of view, although the experimental procedure of deceiving participants should have proceeded with caution, such as the psychological burden on participants, the causal relationship between fraud victimisation and related factors can be determined while ensuring ecological validity.

## Conclusion

The present study clarified the psychosocial characteristics of victims of special fraud among Japanese older adults. The findings of this study can be summarised with the following suggestions for practise in fraud prevention: Because elderly women who live alone and do not go out frequently are often victims of special fraud, they should be monitored by the police station and welfare administration as countermeasures for their fraud victimisation. Moreover, because of common characteristics of these elderly victims such as overconfidence against fraud victimisation and responding quickly to phone calls and unknown visitors prior to becoming victims of special fraud, it is necessary to distribute phones with a recording or detecting function to those who fall under these characteristics and inform their family members and others about what to do and where to go for advice when they receive suspecting phone calls or visitors. Recently, there are integrated ways of detecting frauds in phone calls in Japan; first, the real-time call data is analysed, and if fraud is suspected, the system automatically disconnects calls at once (COCOWADOCO, Inc.); second, the recorded call data is analysed, and if fraud is suspected, the system alerts the call receivers by sending an e-mail or phone call to them or their relatives who have been pre-registered during the call (NTT WEST Corp.). These phone systems do not require the caller to determine whether the call is fraudulent or not. They are effective countermeasures for those who can confidently avoid fraud victimisation and quickly respond to phone calls and for those experiencing cognitive decline.

These countermeasures are not enough if implemented exclusively by the police. Consumer affairs administrations, such as consumer affairs centres, and welfare administrations, such as Community General Support Centres, should work together to defend older adults from fraud, including those experiencing cognitive decline (Ueno et al., 2021). Regional councils for ensuring consumer safety (Consumer Affair Agency, 2015) are not subject to the Act on the Protection of Personal Information concerning consumption-related issues and can share personal information without the individual’s consent. By utilising regional councils for ensuring consumer safety, it is possible to match the information on fraud victims held by the police and consumer affairs administration and the information on dementia and mental and physical disabilities held by the welfare administration, and to integrate individual countermeasures implemented by each administrative agency to provide monitoring and education for individuals who are vulnerable to fraud. In response to the extremely tragic situation of special fraud, where the amount of damage per case is larger than that of theft, it is necessary for administrative agencies such as the police, consumer, and welfare administration should proactively cooperate daily and establish a system to monitor the safety and security of older adults.

## Data availability statement

The raw data supporting the conclusions of this article will be made available by the authors, without undue reservation.

## Ethics statement

The studies involving human participants were reviewed and approved by Medical Ethics Review Committee of Kyoto Prefectural University of Medicine (ERB-C-1845). The patients/participants provided their written informed consent to participate in this study.

## Author contributions

DU: execution of the research project, statistical analysis, writing the first draft, and manuscript review and critique. MA and YF: execution of the research project, data collection, and manuscript review and critique. SA: assistance in the execution of the research project and manuscript review and critique. YK and TM: statistical review and critique, and manuscript review and critique. JN: organisation and execution of the research project, statistical review and critique, and manuscript review and critique. All authors contributed to the article and approved the submitted version.

## Funding

This work was supported by JSPS KAKENHI grant number 22 K13854.

## Acknowledgments

We thank the staff in the Second Organized Crime unit, Kyoto Prefectural Police Headquarter, who recruited the participants and assisted in the research project. We also thank all of the study participants.

## Conflict of interest

The authors declare that the research was conducted without any commercial or financial relationships that could be construed as a potential conflict of interest.

## Publisher’s note

All claims expressed in this article are solely those of the authors and do not necessarily represent those of their affiliated organizations, or those of the publisher, the editors and the reviewers. Any product that may be evaluated in this article, or claim that may be made by its manufacturer, is not guaranteed or endorsed by the publisher.
